# Supplementary material for: Saturated Fatty Acids Promote GDF15 Expression in Human Macrophages through the PERK/eIF2/CHOP Signaling Pathway
Source: Nutrients. 2020 Dec 8;12(12):3771. doi: 10.3390/nu12123771 (PMC7764024; doi:10.3390/nu12123771)
Supplement: Supplementary file 1 [file nutrients-12-03771-s001.pdf]

nutrients

MDPI

Supplementary Materials

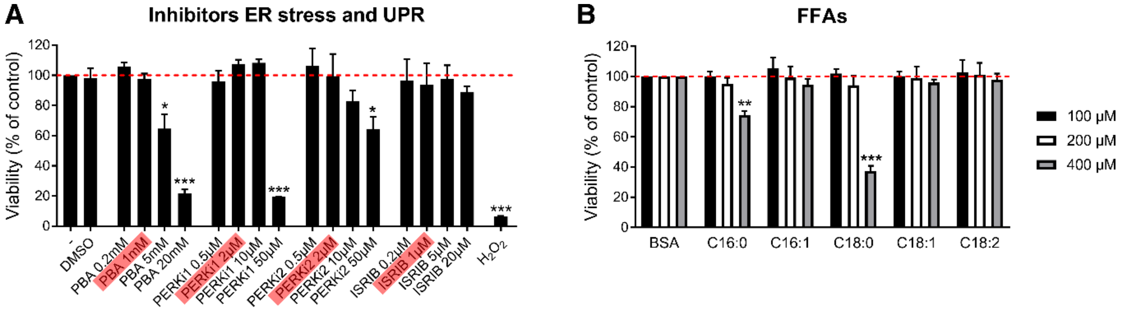

Figure S1. Cell toxicity of inhibitors and FFAs. (A–B) Cell viability was assessed with WST1 reagent in PMA-differentiated THP-1 cells treated for 16 hrs with inhibitors or FFAs at the indicated concentration (n = 3). *p < 0.05, **p < 0.01, ***p < 0.001 by one-way ANOVA with Dunnett's multiple comparisons test for **(A)** and by two-way ANOVA with Sidak's multiple comparisons test for (B). Results are presented as mean ± SEM.

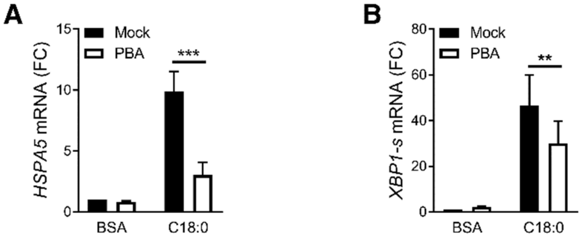

Figure S2. PBA reduces ER stress in MDMs. (A–B) mRNA expression measured by RT-qPCR after stimulation of MDMs with 100 μM C18:0 for 16 hrs in presence of 1 mM PBA (n = 5). **p < 0.01; ***p < 0.001 by two-way ANOVA with Sidak's multiple comparisons test. Results are presented as mean ± SEM. FC, Fold change.

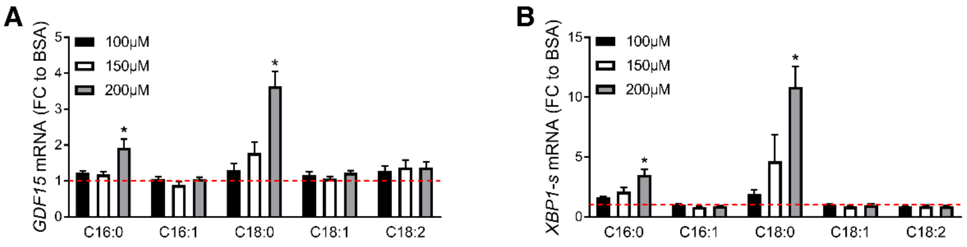

Figure S3. SFAs induce *GDF15* expression and ER stress in PMA-differentiated THP1 cells. (A-B) mRNA expression measured by RT-qPCR after treatment with the indicated concentration of FFAs for 16 hrs in PMA-differentiated THP-1 cells (n = 5). *p < 0.05 by two-way ANOVA with Sidak's multiple comparisons test. Results are presented as mean ± SEM. FC, Fold change.

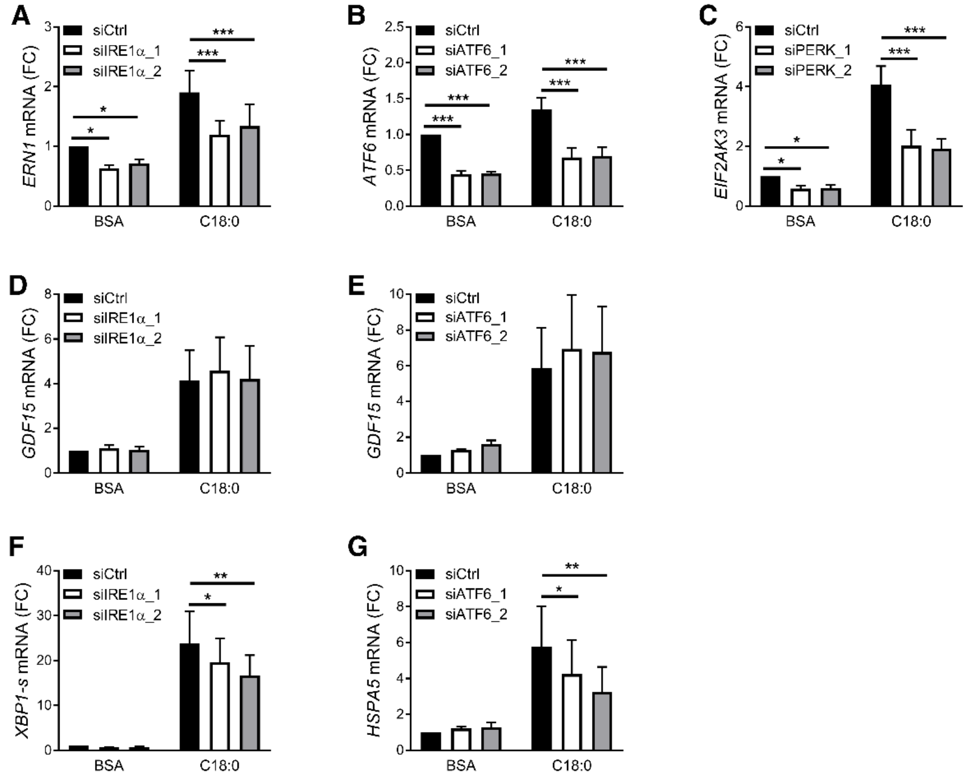

Figure S4. IRE1α, ATF6 and PERK silencing in PMA-differentiated THP1 cells. (A–G) mRNA expression measured by RT-qPCR after treatment with 200 μM C18:0 for 16 hrs in PMA-differentiated THP-1 cells after IRE1α, ATF6 or PERK silencing by siRNA ($n = 5$). *$p < 0.05$, **$p < 0.01$, ***$p < 0.001$ by two-way ANOVA with Sidak's multiple comparisons test. Results are presented as mean ± SEM. FC, Fold change.

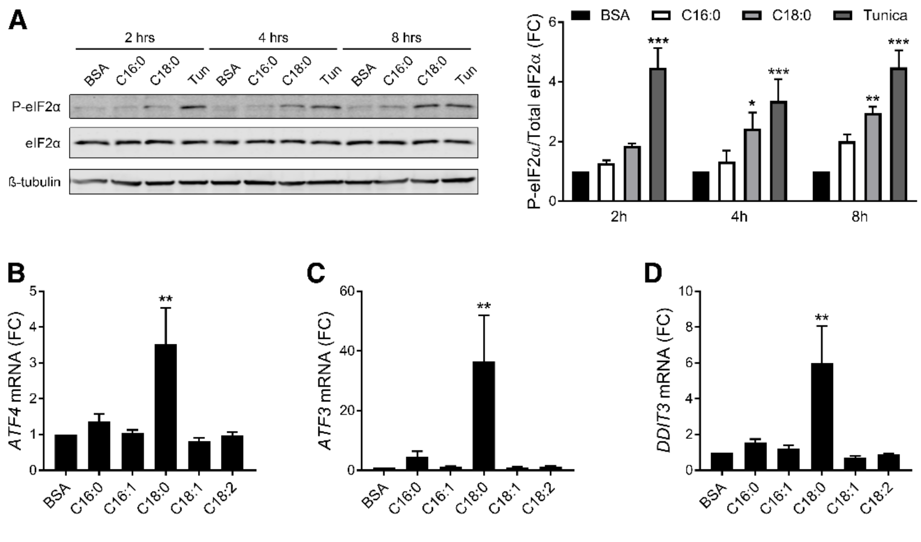

Figure S5. SFAs induce ISR in macrophages. (A) Analysis of eIF2α phosphorylation by western blot and quantification after stimulation of PMA-differentiated THP-1 cells with 200 μM SFAs or 5 μg/mL tunicamycin for 2, 4 or 8 hrs ($n = 5$). (B-D) mRNA expression measured by RT-qPCR after treatment with 100 μM FFAs in MDMs ($n = 5$). *$p < 0.05$, **$p < 0.01$, ***$p < 0.001$ by two-way ANOVA with Sidak's multiple comparisons test for (A) and by one-way ANOVA with Dunnett's multiple comparisons test for (B-D). Results are presented as mean ± SEM. FC, Fold change.

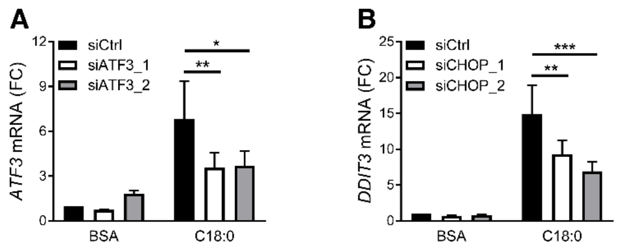

Figure S6. ATF3 and CHOP silencing in PMA-differentiated THP1 cells. (A-B) mRNA expression measured by RT-qPCR after treatment with 200 μM C18:0 for 16 hrs in PMA-differentiated THP-1 cells after ATF3 or CHOP silencing by siRNA (*n* = 4). **p* < 0.05, ***p* < 0.01, ****p* < 0.001 by two-way ANOVA with Sidak's multiple comparisons test. Results are presented as mean ± SEM. FC, Fold change.

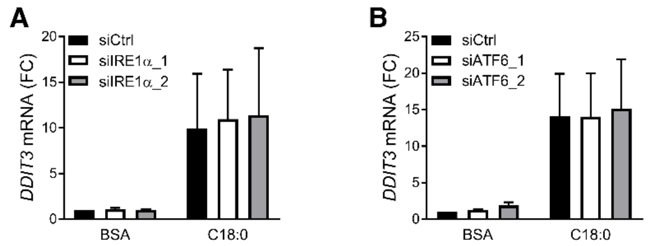

Figure S7. IRE1α and ATF6 silencing on *DDIT3* expression in PMA-differentiated THP1 cells. (A-B) *DDIT3* expression measured by RT-qPCR after treatment with 200 μM C18:0 for 16 hrs in PMA-differentiated THP-1 cells after IRE1α or ATF6 silencing by siRNA (*n* = 5). No statistical differences by two-way ANOVA with Sidak's multiple comparisons test. Results are presented as mean ± SEM. FC, Fold change.

Table S1. Sequences of siRNA.

Target gene	siRNA	Sense	Antisense
ERN1	**siIRE1α_1**	GGACAGGCUCAAUCAAAUGGACUUU	AAAGUCCAUUUGAUUGAGCCUGUCC
ERN1	**siIRE1α_2**	CCGAAGUUCAGAUGGAAUCCUCUAC	GUAGAGGAUUCCAUCUGAACUUCGG
ATF6	**siATF6_1**	CCAAUUAUCAGUUUACAACCUGCAC	GUGCAGGUUGUAAACUGAUAAUUGG
ATF6	**siATF6_2**	AGAAUAUAUGCUAGGGUUAGAGGCG	CGCCUCUAACCCUAGCAUAUAUUCU
EIF2AK3	**siPERK_1**	GGUAAUGCGAGAAGUUAAAGCCUUA	UAAGGCUUUAACUUCUCGCAUUACC
EIF2AK3	**siPERK_2**	GCAGUAUCCAUAUGAUAAUGGUUAU	AUAACCAUUAUCAUAUGGAUACUGC
DDIT3	**siCHOP_1**	AAGUGACUUUCUAUGAGCCAAAAAA	UUUUUUGGCUCAUAGAAAGUCACUU
DDIT3	**siCHOP_2**	GAACCAGCAGAGGUCACAAGCACCU	AGGUGCUUGUGACCUCUGCUGGUUC
ATF3	**siATF3_1**	GCAUUUGAUAUACAUGCUCAACCUU	AAGGUUGAGCAUGUAUAUCAAAUGC
ATF3	**siATF3_2**	CGACGAGAAAGAAAUAAGAUUGCAG	CUGCAAUCUUAUUUCUUUCUCGUCG

Table S2. List of primers used in RT-qPCR.

Gene	Accession numbers	Forward	Tm	Reverse	Tm
**GDF15**	NM_004864	GCAAGAACTCAGGACGGTGA	59.97	TGGAGTCTTCGGAGTGCAAC	59.97
**OAZ1**	NM_004152 ; NM_001301020	GGATCCTCAATAGCCACTGC	58.11	TACAGCAGTGGAGGGAGACC	60.61
**CDKN1A**	NM_000389 ; NM_078467 ; NM_001291549 ; NM_001220778 ; NM_001220777 ; NM_001374509 ; NM_001374510 ; NM_001374511 ; NM_001374512 ; NM_001374513	GCGAGGCACAAGGGTACAAGACAG	65.67	TAATGGCGGGCTGCATCCAG	62.96
**MDM2**	NM_002392 ; NM_001145339 ; NM_001145337 ; NM_001145340 ; NM_001278462 ; NM_001367990	GCCCTTCGTGAGAATTGGCT	60.68	AAAGCCCTCTTCAGCTTGTGT	60.13
**HSPA5**	NM_005347	TAGCGTATGGTGCTGCTGTC	60.18	TTTGTCAGGGGTCTTTCACC	57.64
**XBP1-s**	NM_001079539	CTGAGTCCGCAGCAGGTG	60.43	ACTGGGTCCAAGTTGTCCAG	59.53
**ERN1**	NM_001433	AGCAGTTAGAGAGAGGCGGG	61.04	CTGGAGGGGGACAGTGATGT	60.91
**ATF6**	NM_007348	CAATTGGAAGCAGCAAATGA	55.12	ACCGAGGAGACGAGACTGAA	59.96
**EIF2AK3**	NM_004836 ; NM_001313915	GTGGGACCAAGACCGTGAAA	60.18	CGACAACCCAGAGCTGAACA	60.25
**ATF4**	NM_001675 ; NM_182810	TCAAACCTCATGGGTTCTCC	57.12	GTGTCATCCAACGTGGTCAG	58.85

**DDIT3**	NM_001195053 ; NM_001195054 ; NM_001195055 ; NM_001195056 ; NM_004083 ; NM_001195057	GAACCAGGAAACGGAAACAGA	58.43	TCTCCTTCATGCGCTGCTT	59.70
**ATF3**	NM_001674 ; NM_001030287 ; NM_001040619 ; NM_001206484 ; NM_001206486 ; NM_001206488	ATTGTCCGGGCTCAGAATGG	60.11	ACCACGACTGCTTAGCTCTG	59.76

Table S3. List of primers used in ChIP-qPCR.

Site	Forward	Tm	Reverse	Tm
**Promoter site A**	GCTGGAGCTTGCAGGTTGG	61.63	AAAATTTCAGAAATTAACCGGTCGTG	59.36
**Promoter site B**	ACCACGACCGGTTAATTTCTGAA	60.49	AAAAATTAAAACTCTAGCCCCTGGC	60.05
**Promoter site C**	TGCTCTTGTTGATCAGGCGGA	62.05	CTGGGCTTGGTGGTGGGATTA	62.09
**Promoter site D**	AGAATGAGTAGGAGTTCTCCAGAGG	60.87	CATAGGAGGCACTTGATAAACTGGG	61.21
**Promoter site NS**	ATTATGAATCCTGTGAGGATGGCTT	60.16	ATTATCTTTCCAGTCTAAGCAGGGT	59.57
